# Prior procedures, graft location, preoperative physical health, postoperative strength and graft integrity are associated with 10‐year clinical outcome after matrix‐induced autologous chondrocyte implantation

**DOI:** 10.1002/ksa.12699

**Published:** 2025-07-07

**Authors:** Jay R. Ebert, Peter K. Edwards, Sven Klinken, David J. Wood, Gregory C. Janes

**Affiliations:** ^1^ School of Human Sciences (Exercise and Sport Science) University of Western Australia Perth Western Australia Australia; ^2^ HFRC Rehabilitation Clinic Perth Western Australia Australia; ^3^ School of Allied Health Curtin University Perth Western Australia Australia; ^4^ Perth Radiological Clinic Perth Western Australia Australia; ^5^ School of Surgery (Orthopaedics) University of Western Australia Perth Western Australia Australia; ^6^ Perth Orthopaedic & Sports Medicine Centre Perth Western Australia Australia

**Keywords:** clinical outcomes, matrix‐induced autologous chondrocyte implantation, patient satisfaction, predictors

## Abstract

**Purpose:**

To investigate factors associated with 10‐year clinical scores after matrix‐induced autologous chondrocyte implantation (MACI).

**Methods:**

This retrospective case series included 143 MACI patients with 10‐year clinical and radiological follow‐up. Clinical assessment included the Knee Injury and Osteoarthritis Outcome Score (KOOS) quality of life (KOOS‐QOL) and sport and recreation (KOOS‐Sport/Rec) subscales, and patient satisfaction. Regression analysis investigated the contribution of patient (age, sex, body mass index and preoperative physical health), surgical (symptom duration, prior surgeries, defect size and location) and postoperative variables, such as knee extensor strength and a magnetic resonance imaging (MRI) composite graft score, to 10‐year clinical scores and being ‘very satisfied’.

**Results:**

The KOOS‐QOL (*p* < 0.001) and KOOS‐Sport/Rec (*p* < 0.0001) improved from baseline to 10 years, with 88 (61.5%) patients ‘very satisfied’ with their outcome. A lower number of prior surgical procedures, greater knee extensor strength, and a better 10‐year MRI score contributed significantly to a higher KOOS‐QOL. For the KOOS‐Sport/Rec, a lower number of prior surgical procedures, undergoing tibiofemoral (versus patellofemoral) MACI, greater preoperative physical health and a better 10‐year MRI score contributed significantly to a better score. Finally, a lower number of prior procedures and greater preoperative physical health contributed to a patient being ‘very satisfied’.

**Conclusion:**

Factors including the number of prior procedures, defect location, preoperative physical health, postoperative quadriceps strength and MRI‐based graft integrity were associated with improved 10‐year clinical scores. This information will permit more tailored patient discussion, along with the setting of realistic, longer‐term expectations for outcomes.

**Level of Evidence:**

Level IV.

**Abbreviations**
MACI: matrix‐induced autologous chondrocyte implantation
MRI: magnetic resonance imaging
QOL: quality of life

## INTRODUCTION

Several options exist for the treatment of symptomatic knee cartilage defects, including marrow stimulation techniques, osteochondral autograft transplantation (OAT), osteochondral allograft (OCA) and autologous chondrocyte implantation (ACI), with significant postoperative improvement reported for all procedures [29]. Third‐generation matrix‐induced (MACI) has demonstrated encouraging short‐ and mid‐term clinical outcomes [3, 5, 13, 14, 22, 45]. While published research continues to emerge reporting longer‐term MACI outcomes (≥10 years) [1, 10, 12, 20, 23, 30, 42], these remain limited. Furthermore, the surgeon must consider a range of patient‐specific (such as age and body mass index [BMI]) and defect‐specific (such as size, location and containment) variables in the treatment of cartilage lesions [7]. While published studies report on factors associated with clinical outcome after a range of cartilage procedures [4, 9, 28, 35, 36, 39, 40, 41, 43], these generally have small sample sizes and follow‐up periods within 5–10 years (and often no more than 1–2 years). Few studies have reported factors associated with outcome after third‐generation MACI, and all within 5 years of the procedure [15, 17, 19].

Information is lacking on what factors are associated with a successful longer‐term outcome after cartilage repair surgery, let alone MACI. This information would permit tailored preoperative patient discussion, along with setting realistic, longer‐term expectations. This study sought to investigate which patient and surgical characteristics, as well as postoperative variables, are associated with pertinent 10‐year clinical outcomes (quality of life and activity) and satisfaction after MACI. First, it was hypothesised that clinical outcomes for the cohort would significantly improve from baseline (presurgery) to 10 years postsurgery. Second, it was hypothesised that certain patient demographics, defect and surgical characteristics, and postoperative variables, would be associated with 10‐year reported: (1) quality of life, (2) sport and recreation capacity and (3) being very satisfied with their overall outcome.

## MATERIALS AND METHODS

**Figure 1:**
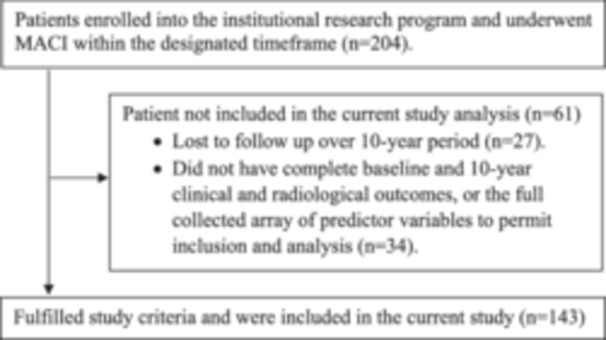
Flowchart demonstrating initial numbers of patients undergoing matrix‐induced autologous chondrocyte implantation (MACI) and enroled into the institutional research programme, along with those that fulfilled the current study criteria, including having baseline and 10‐year postoperative clinical and radiological outcomes, as well as full documentation of all predictor variables employed.

Overall, 143 patients were included in the current analysis (Figure 1). Patients underwent MACI between December 2003 and June 2013 for a symptomatic cartilage lesion in the tibiofemoral (TF, *n* = 96) or patellofemoral (PF, *n* = 47) joint, undertaken by one of five orthopaedic surgeons. The indication for MACI during that period included patients presenting with symptomatic, full‐thickness grade III or IV chondral lesions as per the International Cartilage Repair Society classification system [6]. Patients were 15–65 years of age and, as part of the current study, this included 99 patients (69%) who were <39 years of age and 44 patients (31%) who were ≥40 years of age. Patients presenting with joint malalignment underwent MACI and were included if they underwent concomitant offloading osteotomy (*n* = 4) in the presence of significant varus/valgus lower limb deformity (as indicated by >3° tibiofemoral anatomic angle) in cases of TF defects, or Fulkerson osteotomy (*n* = 16) in those with PF malalignment (assessed via Computed Tomography imaging and >0.9 cm lateralisation of tibial tuberosity) in cases of PF defects. Other concomitant procedures undertaken at the time of MACI included anterior cruciate ligament (ACL) reconstruction (*n* = 5), posterior cruciate ligament (PCL) reconstruction (*n* = 1), isolated lateral release (*n* = 6) and partial meniscectomy (*n* = 6). All patients were recruited into an institutional research programme prior to their MACI surgery, with informed consent obtained as per the institutional Human Research Ethics Committee (HREC).

### MACI surgical procedure

All patients underwent third‐generation MACI, whereby harvested and cultured cells were seeded onto a type I/III collagen membrane (ACI‐Maix Matricel GmbH), and subsequently adhered to the subchondral bone underlying the chondral defect via fibrin glue. Rehabilitation was tailored to patients based on graft location, concomitant surgeries and the individual patient's conditioning and tolerance to exercise, with protocols previously reported [12, 16, 21].

### Clinical outcome measures

First, the Knee Injury and Osteoarthritis Outcome Score (KOOS) was employed and is a knee‐specific questionnaire including five individual subscales: pain, symptoms, activities of daily living, sport and recreation and quality of life, with each of these subscales scored from 0 (worst) to 100 (best) [34]. This patient‐reported survey has been recommended for use in patients undergoing cartilage repair [32]. For the current study, the 10‐year KOOS‐quality of life (QOL) and KOOS‐sport and recreation (Sport/Rec) subscales were included and have been reported as outcome measures responsive to change after surgery [17]. The 10‐year patient acceptable symptomatic state (PASS) thresholds after third‐generation ACI have been reported as 59.4 (KOOS‐QOL) and 57.5 (KOOS‐Sport/Rec) [25]. Second, all patients completed a satisfaction questionnaire at 10 years postsurgery, which has been previously employed [10, 12]. For the current study, the survey asked patients to rate their level of satisfaction with their surgical outcome overall. A Likert response scale with descriptors ‘very satisfied’, ‘somewhat satisfied’, ‘somewhat dissatisfied’ and ‘very dissatisfied’ was employed. Predictor variables outlined below were used to investigate the association between being ‘very satisfied’ versus any of the other responses (‘somewhat satisfied’, ‘somewhat dissatisfied’ and ‘very dissatisfied’).

### Predictor variables

A range of variables were selected for inclusion in the predictive models, dictated first by what had been reported in other cartilage repair studies [4, 9, 15, 17, 19, 28, 35, 36, 39, 40, 41], although second by what was available based on collection in the current cohort. Patient demographics selected included age, sex and BMI. Preoperative injury/surgery characteristics included duration of symptoms (DOS), the number of knee surgeries prior to undergoing MACI (not including the first‐stage cartilage biopsy, unless another procedure was indeed undertaken at that time), defect size and defect/graft location (TF or PF). Preoperative general health was included and assessed via the 36‐item Short Form Health Survey (SF‐36) and included both mental (MCS) and physical (PCS) component subscales. Finally, postoperative 10‐year variables that were selected included the peak isokinetic knee extensor (quadriceps) torque (normalised to body weight) and a magnetic resonance imaging (MRI) composite score. Peak isokinetic knee extensor torque was assessed at an angular velocity of 90°/s at 10 years using an isokinetic dynamometer (Isosport International, Gepps Cross). The MRI Composite Score for each patient was assessed using their 10‐year MRI. Eight pertinent parameters of graft repair (graft infill, signal intensity, border integration, surface contour, tissue structure, effusion, subchondral lamina and bone) were assessed [27], as per the magnetic resonance observation of cartilage repair tissue (MOCART) system [26, 31, 38, 44]. Each parameter was scored individually from 1 to 4 (1 = poor; 2 = fair; 3 = good; 4 = excellent) in comparison to the adjacent native cartilage. The MRI composite score was then calculated by multiplying each individual score by a weighting factor [31] and summing scores [11].

All 10‐year postoperative MRI scans were scored by a single independent, experienced musculoskeletal radiologist with extensive experience employing the MOCART scoring tool since its inception and initial publication. Intraobserver reliability was evaluated for the eight pertinent morphological MRI scores (kappa coefficient), as well as the continuous MRI composite score (intraclass correlation coefficient), by re‐scoring a random selection of 20. Significant correlations were observed for the MRI composite score (*ρ* = 0.811) and each of the eight graft‐related scoring parameters (graft infill *ρ* = 0.949; signal intensity *ρ* = 1.00; border *ρ* = 0.982; surface contour *ρ* = 1.00; structure *ρ* = 0.840; subchondral lamina *ρ* = 1.00; subchondral bone *ρ* = 0.920; effusion *ρ* = 0.993).

### Statistical analysis

Descriptive statistics were performed to report baseline clinical scores, patient and surgical characteristics and 10‐year clinical scores. Normality was assessed using the Shapiro–Wilk test. Paired sample *t*‐tests were used to estimate the degree of change in clinical outcomes from before MACI to 10 years after surgery for normally distributed data, and the Wilcoxon Signed‐Rank test for data not normally distributed. Furthermore, frequency counts were performed to show the proportion of patients who met a >10‐point difference in KOOS outcomes, defining the minimum cut‐off for a clinically significant difference [33]. The Pearson correlation coefficient (or Spearman rho [*ρ*] in the case of nonparametric data) was used to quantify the association between outcome measures. Univariable and multivariable linear regressions were employed for continuous dependent variables, namely 10‐year KOOS‐QOL and KOOS‐Sport/Rec, while binary logistic regression analyses were employed for 10‐year overall satisfaction (‘very satisfied’ versus all other satisfaction responses) with the surgery. The aim was to examine the *a priori* hypothesised association of certain patient and surgical variables with clinical outcomes 10 years post‐MACI. For each regression model, potential predictors were first evaluated univariably, and those displaying associations with outcomes at *p* < 0.10 were included in a multivariable regression model for the relevant clinical outcome. In the final step, nonsignificant variables (*p* < 0.05) were removed from the initial multivariable model one at a time, while making sure that remaining coefficients did not change by more than 20% to ensure the retention of important confounders in the model, as recommended by Hosmer et al. [24]. In the linear regression model for KOOS‐QOL and KOOS‐Sport/Rec, Beta coefficients, along with their 95% confidence intervals (CI), were calculated to quantify the influence of each predictor on the dependent variable, with standardised Beta values (*β*) included in the final multivariable model further indicating the importance of each predictor. For the binary logistic regression pertaining to overall satisfaction with the surgery, each predictor variable was expressed as odds ratios (OR) and 95% CI. These were used to evaluate the relationship between each predictor variable and the dependent variable. The Nagelkerke *R* ^2^ was employed to compare the performance of the predictor variables, serving also as an overall performance measure for the model (explained variation). The Hosmer–Lemeshow goodness‐of‐fit test was used to examine the model's calibration, focusing specifically on the congruity between predictions and observed outcomes. All analyses were carried out using SPSS software (SPSS, Version 29.0, SPSS Inc).

## RESULTS

Baseline scores, patient and surgical characteristics, and 10‐year scores are shown (Table 1). Due to non‐normality of the KOOS‐QOL and KOOS‐Sport/Rec (Shapiro–Wilk test, *p* < 0.001), a Wilcoxon Signed‐Rank test was used. Mean improvement in KOOS‐QOL from baseline was 35.6 points (95% CI, 31.9–39.3; *p* < 0.001), and 122 of 143 (85.3%) patients had an improvement of ≥10 points. For the KOOS‐Sport/Rec, a 41.5 point mean improvement was observed (95% CI, 36.8–46.1; *p* < 0.001), with 125 of 143 (87.4%) patients demonstrating improvement ≥10 points. Overall, 88 (61.5%) patients were ‘very satisfied’ with their 10‐year outcome.

**Table 1:** Descriptive statistics of baseline patient and surgical characteristics and clinical scores, as well as 10‐year clinical outcome scores in 143 patients.

	Mean ± SD or *n* (%)	
	Baseline	10 years postsurgery
Participant characteristics		
Age, years	36.6 ± 10.6	
Sex—female, *n* (%)	53 (37.1%)	
BMI, kg/m^2^	26.05 ± 3.11	
Duration of symptoms, months	7.93 ± 6.93	
Prior surgical procedures, *n*	1.15 ± 1.11	
Surgical characteristics		
Defect size (cm^2^)	3.25 ± 1.92	
Location—tibiofemoral (vs. patellofemoral)	96 (67.1%)	
Clinical outcome		
KOOS‐QOL	28.92 ± 19.71	64.51 ± 21.84
KOOS‐Sport/Rec	27.13 ± 23.82	68.59 ± 24.64
SF‐36 MCS	51.75 ± 8.78	55.29 ± 7.30
SF‐36 PCS	37.34 ± 9.50	49.27 ± 9.09
Knee extensor torque, Nm/kg^−1^		2.00 ± 0.75
Satisfaction—very satisfied (vs. other responses), *n* (%)		88 (61.5%)
MRI composite score		2.99 ± 0.61

Abbreviations: BMI, body mass index; kg/m^2^, kilograms per metre squared; KOOS, Knee Injury and Osteoarthritis Outcome Score; MCS, mental component subscale; MRI, magnetic resonance imaging; Nm/Kg, Newton‐metres per kilogram; PCS, physical component subscale; QOL, quality of life; SF‐36, 36‐item Short Form Health Survey; Sport/Rec, sport and recreation.

### KOOS‐QOL

The number of prior surgical procedures, knee extensor strength, and the 10‐year MRI score contributed significantly to KOOS‐QOL in the final multivariable model (Table 2). For every additional prior surgical procedure a patient had undergone prior to MACI surgery, the 10‐year KOOS‐QOL score decreased by 6.07 points (95% CI, −8.81 to −3.32, *p* = 0.019). Knee extensor strength was also predictive of 10‐year KOOS‐QOL scores, in that for every one‐unit increase in the knee extensor torque, the 10‐year KOOS‐QOL increased by 5.29 points (95% CI, 1.17 to 9.40, *p* = 0.012). Lastly, for every one‐point increase in the MRI composite score, the 10‐year KOOS‐QOL score increased by 5.49 units (95% CI, 0.52 to 10.49, *p* = 0.023). The standardised *β*s (change in units of SD of the KOOS‐Sport/Rec score for a 1‐SD increase in predictor) were 0.346, −0.309, 0.182 and 0.153, respectively. The adjusted *R* ^2^ for this model was 0.305.

**Table 2:** Univariable and multivariable linear regression models for the 10‐year KOOS QOL score.

Predictor variable	Univariable		Multivariable (Adjusted *R* ^2^ = 0.300)		Final multivariable model (Adjusted *R* ^2^ = 0.305)		
	*B* (95% CI)	*p* value	*B* (95% CI)	*p* value	*B* (95% CI)	Standardised *β*	*p* value
Baseline KOOS‐QOL	0.47 (0.30 to 0.63)	<0.001	0.28 (0.14 to 0.41)	<0.001	0.38 (0.23 to 0.54)	0.346	<0.001
Age, years	−0.04 (−0.36 to 0.27)	0.783					
Sex—female	−1.18 (−8.00 to 5.65)	0.734					
BMI, kg/m^2^	0.21 (−0.64 to 1.05)	0.634					
Duration of symptoms, months	−0.48 (−0.95 to 0.01)	0.047	−0.02 (−0.46 to 0.51)	0.930			
Prior procedures, *n*	−6.10 (−8.92 to −3.27)	<0.001	−6.11 (−9.05 to −3.17)	<0.001	−6.07 (−8.81 to −3.32)	−0.309	0.019
Defect size, cm^2^	−1.31 (−3.03 to 0.41)	0.135					
Defect location (TF vs. PF)	−0.28 (−12.13 to 3.23)	0.938					
SF‐36 MCS	−0.19 (−0.58 to 0.20)	0.341					
SF‐36 PCS	0.12 (−0.30 to 0.54)	0.581					
Knee extensor torque, Nm/kg^−1^	4.80 (0.37 to 9.23)	0.034	5.34 (1.05 to 9.62)	0.015	5.29 (1.17 to 9.40)	0.182	0.012
MRI composite score	5.78 (−0.44 to 11.12)	0.034	5.50 (0.51 to 10.50)	0.031	5.49 (0.52 to 10.49)	0.153	0.023

Abbreviations: BMI, body mass index; CI, confidence interval; cm, centimetres; kg/m^2^, kilograms per metre squared; KOOS, Knee Injury and Osteoarthritis Outcome Score; MCS, mental component subscale; MRI, magnetic resonance imaging; Nm/kg, Newton‐metres per kilogram; PCS, physical component subscale; PF, patellofemoral; QOL, quality of life; SF‐36, 36‐item Short Form Health Survey; TF, tibiofemoral.

### KOOS‐Sport/Rec

The number of prior procedures, the location of the defect, the preoperative SF‐36 PCS and the 10‐year MRI composite score contributed significantly to the KOOS‐Sport/Rec in the final multivariable model (Table 3). Specifically, a one‐point increase in the preoperative SF‐36 PCS predicted a 0.66 point increase in the mean 10‐year KOOS‐Sport/Rec (95% CI, 0.24 to 1.08; *p* = 0.002). Patients with a PF defect had a 9.35‐point lower KOOS‐Sport/Rec score at 10 years compared to those with a defect in the TF joint (95% CI, −16.84 to −1.87; *p* > 0.001). For each one‐point increase in the MRI composite score, the 10‐year KOOS‐Sport/Rec score increased by 7.81 units (*B* = 7.81, *p* = 0.008). The standardised *β*s (change in units of standard deviation [SD] of the KOOS‐Sport/Rec score for a 1‐SD increase in predictor) were 0.196, −0.303, −0.179, 0.253, and 0.193, respectively. The adjusted *R* ^2^ for this model was 0.246.

**Table 3:** Univariable and multivariable linear regression models for the 10‐year KOOS sport and recreation score.

Predictor variable	Univariable		Multivariable (Adjusted *R* ^2^ = 0.293)		Final multivariable model (Adjusted *R* ^2^ = 0.288)		
	*B* (95% CI)	*p* value	*B* (95% CI)	*p* value	*B* (95% CI)	Standardised *β*	*p* value
Baseline KOOS‐sport/rec	0.331 (0.17 to 0.49)	<0.001	0.18 (0.01 to 0.35)	0.037	0.20 (0.04 to 0.37)	0.196	0.016
Age, years	−0.304 (−0.67 to −0.06)	0.104					
Sex—female	−0.94 (−9.20 to 7.30)	0.821					
BMI, kg/m^2^	−0.13 (−1.13 to 0.87)	0.798					
Duration of symptoms, months	−0.35 (−0.91 to −0.21)	0.216					
Prior procedures, *n*	−6.93 (−10.24 to −3.63)	<0.001	−6.71 (−9.81 to −3.62)	<0.001	−6.70 (−9.80 to −3.60)	−0.303	<0.001
Defect size, cm	−0.66 (−2.69 to 1.38)	0.508					
Defect location (TF vs. PF)	−7.13 (−18.39 to −2.15)	0.014	−8.13 (−15.79 to −0.48)	0.037	−9.35 (−16.84 to −1.87)	−0.179	0.015
SF‐36 MCS	−0.15 (−0.60 to 0.31)	0.528					
SF‐36 PCS	0.67 (0.22 to 1.13)	0.004	0.64 (0.22 to 1.06)	0.003	0.66 (0.24 to 1.08)	0.253	0.002
Knee extensor torque, Nm/kg^−1^	5.07 (0.23 to 10.38)	0.061	3.48 (1.39 to 8.36)	0.160			
MRI composite score	6.32 (−0.01 to 12.63)	0.050	7.81 (2.09 to 13.53)	0.008	7.81 (2.06 to 13.55)	0.193	0.008

Abbreviations: BMI, body mass index; CI, confidence interval; cm, centimetres; kg/m^2^, kilograms per metre squared; KOOS, Knee Injury and Osteoarthritis Outcome Score; LSI, limb symmetry index; MCS, mental component score; MRI, magnetic resonance imaging; Nm/kg, Newton‐metres; PCS, physical component score; PF, patellofemoral; SF‐36, 36‐item Short Form Health Survey; Sport/Rec, sport and recreation; TF, tibiofemoral.

### Patient satisfaction

The number of prior procedures, the 10‐year MRI composite score, the KOOS‐Sport/Rec score and SF‐36 PCS were all factors univariably associated with overall satisfaction (Table 4). The final model consisted of the number of surgical procedures prior to MACI and the SF‐36 PCS. Specifically, for each one‐unit increase in the SF‐36 PCS score, the odds of being ‘very satisfied’ increased by 13% (OR = 1.13, *p* < 0.001). For every additional prior procedure, the odds of being ‘very satisfied’ decreased by 39% (OR = 0.61, *p* = 0.008) (Table 4). Together, these predictors accounted for 34% of the variance in overall satisfaction with the surgery at 10 years (Nagelkerke *R* ^2^ = 0.342). The Hosmer–Lemeshow goodness‐of‐fit test demonstrated that the model fit the data adequately (*χ* ^2^ = 6.90, *p* = 0.547).

**Table 4:** Univariable (conditioning on baseline score) and multivariable binary logistic regression models for 10‐year overall ‘complete’ satisfaction with the MACI procedure.

Predictor variable	Univariate		Multivariable (Nagelkerke *R* ^2^ = 0.389)		Final model (Nagelkerke *R* ^2^ = 0.342)	
	OR (95% CI)	*p* value	OR (95% CI)	*p* value	OR (95% CI)	*p* value
Age, years	0.99 (0.96 to 1.02)	0.388				
Sex—female	1.36 (0.67 to 2.75)	0.397				
BMI, kg/m^2^	1.01 (0.92 to 1.10)	0.875				
Duration of symptoms, months	0.96 (0.92 to 1.01)	0.121				
Prior procedures, *n*	0.50 (0.36 to 0.71)	<0.001	0.63 (0.43 to 0.92)	0.016	0.61 (0.43 to 0.88)	0.008
Defect cize, cm	0.93 (0.78 to 1.10)	0.388				
Defect location (TF vs. PF)	0.68 (0.33 to 1.38)	0.286				
SF‐36 MCS	1.00 (0.95 to 1.05)	0.946				
SF‐36 PCS	1.14 (1.08 to 1.20)	<0.001	1.11 (1.04 to 1.18)	0.001	1.13 (1.07 to 1.19)	<0.001
KOOS‐sport and recreation	1.04 (1.02 to 1.06)	<0.001	1.02 (0.99 to 1.04)	0.151		
Knee extensor torque, Nm/kg^−1^	1.36 (0.85 to 2.15)	0.789				
MRI composite score	1.76 (1.0 to 3.09)	0.049	1.86 (0.97 to 3.56)	0.061		

Abbreviations: BMI, body mass index; CI, confidence interval; cm, centimetres; kg/m^2^, kilograms per metre squared; KOOS, Knee Injury and Osteoarthritis Outcome Score; LSI, limb symmetry index; MACI, matrix‐induced autologous chondrocyte implantation; MCS, mental component score; MRI, magnetic resonance imaging; Nm/kg, Newton‐metres; OR, odds ratio; PCS, physical component score; PF, patellofemoral; SF‐36, 36‐item Short Form Health Survey; Sport/Rec, sport and recreation; TF, tibiofemoral.

## DISCUSSION

The most important findings of the current study were that the number of prior procedures, graft location, preoperative physical health, postoperative quadriceps strength and MRI‐based graft integrity were associated with 10‐year satisfaction, quality of life, and sport and recreation.

In support of the first hypothesis, PROMs significantly improved from baseline to 10 years. Studies have reported longer‐term clinical improvement after third‐generation MACI [1, 10, 12, 20, 23, 30]. Specific to the clinical scores reported in this study, 85% and 87% of patients reported a ≥10‐point improvement in the KOOS‐QOL and KOOS‐Sport/Rec, respectively, reported as a minimum cut‐off representing a clinically significant difference [33]. Furthermore, the 10‐year KOOS scores observed were beyond previously reported PASS thresholds [25]. These outcomes are also reflected by the satisfaction scores reported, with almost 90% of patients satisfied or very satisfied with their outcome.

Variables that were associated with higher knee‐related QOL included a lower number of prior surgical procedures, greater knee extensor strength, and a better 10‐year MRI composite score. Like that observed for the KOOS‐QOL, a lower number of prior surgical procedures and a better 10‐year MRI score were associated with higher KOOS‐Sport/Rec. However, other factors, including the location of the chondral defect and preoperative SF‐36 PCS, were also predictors. Finally, a lower number of prior procedures and higher SF‐36 PCS were most predictive of being ‘very satisfied’. A study investigating predictors of 5‐year clinical outcome reported that factors such as duration of symptoms, graft size, early postoperative weight‐bearing status and preoperative SF‐36 scores were predictors of MRI‐based outcome and/or patient satisfaction at 5 years [17]. Subsequently, other studies have reported that age, gender and duration of symptoms were associated with postoperative activity level [15], while the restoration of knee extensor strength symmetry has been shown to be associated with patient satisfaction in recreational and sporting ability out to 5 years after third‐generation MACI [19]. Finally, a recent study presenting outcomes at a mean of 8.1 years after third‐generation ACI (Novocart 3D; TETEC) reported that BMI and the number of prior knee surgeries were most influential to postoperative clinical scores [43].

With respect to earlier generations of ACI, McNickle et al. [28] reported that patient age and receiving workers' compensation were independent variables predictive of Lysholm scores at a mean of 4.3 years after first‐generation periosteal‐covered ACI. Bhosale et al. [4] investigated the patient‐related variables that were associated with clinical outcome in patients at a mean of 5 years following first (periosteal covered) or second (collagen covered) generation ACI. They reported that higher age, female gender, larger defect size, a smaller number of previous operations and having a defect on the trochlea, lateral femoral condyle, or at multiple locations were associated with a higher benefit. Dugard et al. [9] investigated predictors of successful treatment outcome after first (periosteal covered) or second (collagen covered) generation ACI. They reported that age at surgery, multiple prior operations, defects on the patella and lower preoperative Lysholm scores were associated with re‐intervention of ACI, while age, gender, location and number of defects, number of prior operations and preoperative Lysholm score influenced the likelihood of progressing to arthroplasty. A retrospective review in patients that underwent ACI in the United States between the years 2010 and 2020 reported that older age and tobacco use were associated with an increased rate of conversion from ACI to knee arthroplasty, while being male (versus female) was associated with decreased overall re‐operation rates [2]. Admittedly, third‐generation MACI was only introduced in the United States in 2017. Finally, a recent study in a mixed ACI cohort reported that a higher short‐term Lysholm score was associated with higher preoperative scores, lower age and lateral femoral grafts, with long‐term Lysholm scores associated with a milder defect grade [37].

However, further evidence has grown with respect to other cartilage repair procedures, albeit limited with respect to outcomes at 10 years or beyond. At a minimum 2 years after OCA transplantation, Wang et al. [40] reported that being male and having a higher number of prior procedures were predictors of failure. At a minimum 1‐year after microfracture or mosaicplasty, Solheim et al. [36] reported that factors predictive of a good to excellent outcome included a small defect size and single lesions, normal appearing surrounding cartilage, a high preoperative Lysholm score, young age and a short duration of symptoms, and noninvolvement of the patellofemoral joint. In a systematic review, Smoak et al. [35] reported age as a predictor of successful outcomes and repair longevity after microfracture in the PF joint. At a mean of 5.7 years after microfracture, Weber et al. [41] reported that patients with a BMI > 30 kg/m² reported lower postoperative clinical scores, while patients with isolated tibial plateau defects or multiple defects demonstrated reduced improvements in symptoms. Larger defect size and prior knee surgery were independent risk factors for additional knee surgery following the microfracture procedure. A recent systematic review published by van Tuijn et al. [39] reported that at a minimum of 1 year after microfracture, a less favourable outcome was associated with higher age, larger lesion size, longer duration of symptoms and prior surgery.

While none of the aforementioned studies appear to report on the association between these factors and longer‐term (10 years or beyond) outcome, let alone after third‐generation ACI, factors more commonly identified have included the amount of prior surgeries [9, 40] or preoperative duration of preoperative symptoms [17, 36, 39], defect or graft size [17, 36, 39], preoperative clinical scores [17], age [2, 9, 28, 35, 36, 39] and gender [2, 9, 40], and patients with patellofemoral pathologies [9, 36]. While the current study sought to investigate any association between many of these variables and 10‐year clinical outcome and satisfaction, as opposed to graft failure or need for re‐operation, factors that were identified by the current study aligned with existing studies, included a lower number of prior procedures, PF involvement, and preoperative clinical scores. Other less frequently reported variables identified by the current study included a better 10‐year MRI composite score and greater knee extensor strength. A prior study reported limited correlation between clinical and radiological outcomes at 5 years after MACI [18]. However, it is logical that a better integrated graft and greater quadriceps strength may create a less symptomatic and higher functioning knee, hence the association between a better MRI score and better KOOS‐QOL, KOOS‐Sport/Rec, and satisfaction scores, as well as the association between greater quadriceps strength and a higher knee‐related QOL.

Some limitations should be acknowledged. First, while the MRI Composite score was employed as a factor within the models, the current study sought to investigate the association between various factors and 10‐year patient‐reported outcomes and satisfaction scores. These are psychosocial constructs that can be influenced by a wide array of variables not assessed, while future research may seek to evaluate what factors are predictive of a successful MRI‐based outcome and/or need for re‐operation. Second, the upper age limit of patients undergoing MACI in the current study was 65 years. It should be acknowledged that this is beyond the current recommended practice, although a reflection of the age indication used at the time that patients were recruited for the current study (i.e., recruitment was from December 2003). Of interest is that the recently published work has reported long‐term success when employing third‐generation ACI when treating knee cartilage lesions in the presence of knee osteoarthritis [8]. Third, other factors that have been reported were not collected at the time of patient recruitment and, therefore, could not be included, such as workers' compensation status [28] or tobacco use [2]. Furthermore, while knee joint compartment (TF or PF) was included, specific graft location (i.e., medial or femoral condyle, patella or trochlea) or defect aetiology (dislocation, nontraumatic and degenerative lesions) could not be included, given the numbers did not permit it (specific location) or detail was not accurately collected (defect aetiology). Given the nature of the study, we were not able to obtain an accurate assessment of early rehabilitation diligence and adherence, or even sports participation or any other knee injuries throughout the 10‐year postoperative timeline. While these variables would be difficult to accurately assess anyway, they were not included in the current study analysis. Finally, we employed KOOS subscales that we deemed relevant to this patient population, which have been recommended for use in patients undergoing cartilage repair [32]. However, other PROMs have been employed, and future research may detect significant influence of other factors dictated by the nature of the PROM.

## CONCLUSION

Factors including the number of prior procedures, defect location, preoperative physical health, postoperative knee extensor strength and MRI‐based graft integrity were associated with improved 10‐year clinical scores. This information will permit more tailored discussion with patients, along with the setting of realistic, longer‐term expectations for outcomes.

## AUTHOR CONTRIBUTIONS

Jay R. Ebert, Peter K. Edwards, David J. Wood and Gregory C. Janes conceived and designed the study. Jay R. Ebert, Peter K. Edwards and David J. Wood supervised the conduct of the study. Jay R. Ebert, Peter K. Edwards and Sven Klinken analysed the data. Jay R. Ebert and Peter K. Edwards wrote the initial drafts. All authors critically revised the manuscript and ensured the accuracy of the data and analysis. All authors have seen and agree with the contents of the manuscript and agree that the work has not been submitted or published elsewhere in whole or part.

## CONFLICT OF INTEREST STATEMENT

The authors declare no conflicts of interest.

## ETHICS STATEMENT

This research was approved by the Hollywood Private Hospital (HPH145) Human Research Ethics Committee (HREC). Informed and written consent was obtained from all individual participants included in the study.

## Data Availability

Data have not been made publicly available, although data sets generated during the current study can be made available from the corresponding author upon reasonable request.
